# Supplementary material for: Growth of bilayer MoTe2 single crystals with strong non-linear Hall effect
Source: Nat Commun. 2022 Sep 17;13:5465. doi: 10.1038/s41467-022-33201-3 (PMC9482631; doi:10.1038/s41467-022-33201-3)
Supplement: Supplementary file 1 — Supplementary information [file 41467_2022_33201_MOESM1_ESM.pdf]

Supplementary Information for

Growth of Bilayer $MoTe_2$ Single Crystals with Strong Non-Linear Hall Effect

Teng Ma[1,2#], Hao Chen[1,3#], Kunihiro Yananose[4#], Xin Zhou[1], Lin Wang[1], Runlai Li[1], Ziyu Zhu[1], Zhenyue Wu[1], Qing-Hua Xu[1], Jaejun Yu[4], Cheng Wei Qiu[5], Alessandro Stroppa[6]*, and Kian Ping Loh[1,2,3]*

[1]Department of Chemistry, National University of Singapore, Singapore 117543, Singapore.

[2]Department of Applied Physics, Hong Kong Polytechnic University, Hung Hom, Kowloon, Hong Kong, P. R. China.

[3]Centre for Advanced 2D Materials, National University of Singapore, 6 Science Drive 2, Singapore 117546.

[4]Center for Theoretical Physics, Department of Physics and Astronomy, Seoul National University, Seoul 08826, Republic of Korea.

[5]Department of Electrical and Computer Engineering, National University of Singapore, Singapore 117583

[6]Consiglio Nazionale delle Ricerche, Institute for Superconducting and Innovative Materials and Devices (CNR-SPIN), c/o Department of Physical and Chemical Sciences, University of L'Aquila, Via Vetoio I-67100 Coppito, L'Aquila, Italy.

[#]These authors contributed equally to this work.

*Correspondence to chmlohkp@nus.edu.sg, alessandro.stroppa@spin.cnr.it

Supplementary Figures.

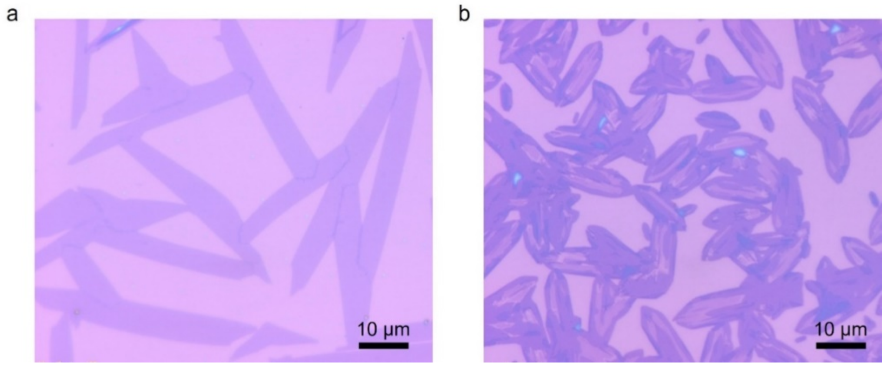

Supplementary Figure 1. Optical images of $MoTe_2$ films grown with different H_2 concentration. a, Monolayer dominated $MoTe_2$ single crystals with sharp edges and regular rectangular shapes grown under the H_2/Ar flow rate of 10/200, suggesting the attachment-limited growth mechanism. **b**, Monolayered and few-layered $MoTe_2$ single crystals with rounded edges and irregular shapes grown under H_2/Ar flow rate of 30/200, suggesting that the growth mechanism changed to be diffusion-limited due to the increased H_2 concentration.

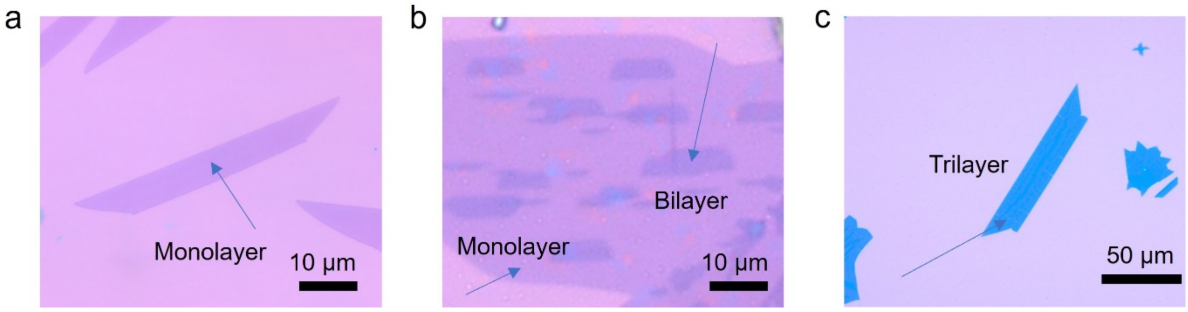

Supplementary Figure 2. Fabrication process of trilayer $MoTe_2$ single crystals. a, Optical image of initial monolayer $MoTe_2$ single crystal. **b**, Optical image of aligned monolayer $MoTe_2$ single crystal on top of the monolayer. **c**, Optical image of the large-scale trilayer $MoTe_2$ single crystal.

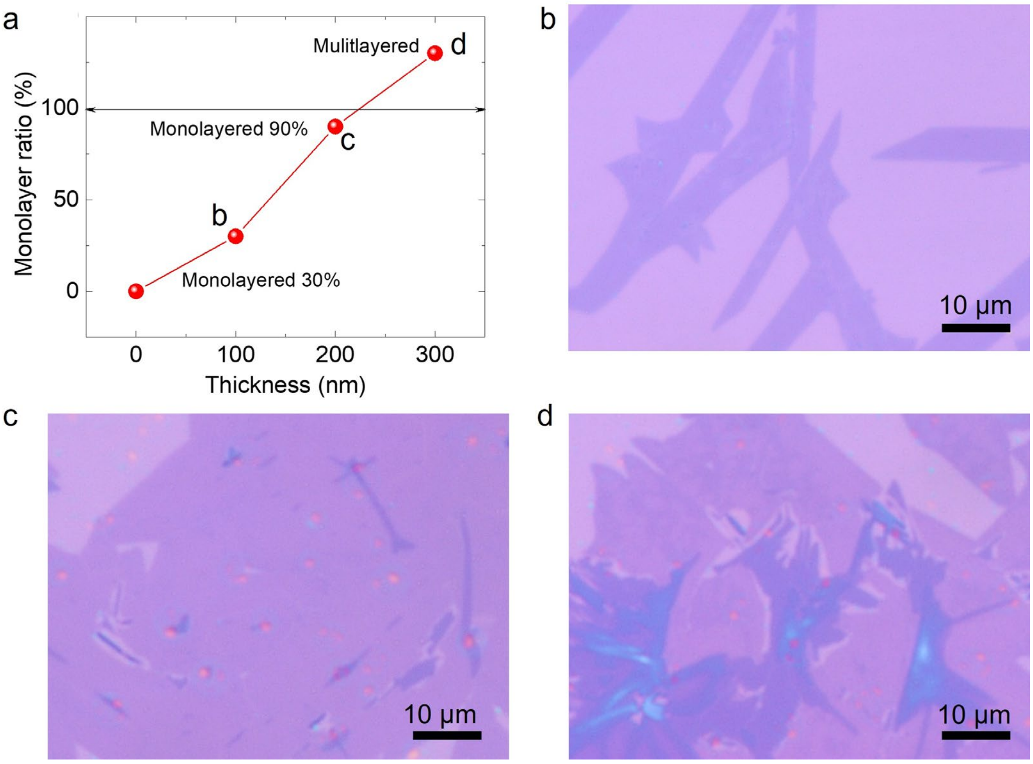

Supplementary Figure 3. Optical images of $MoTe_2$ films grown with different thickness of the AHM precursor. a, The monolayer ratio as a function of the thickness of the AHM precursor. **b-d**, Optical images of monolayer coverage for 30% (**b**), 90% (**c**), and multi-layered (**d**) $MoTe_2$ fabricated by using the thickness of 100 nm (**b**), 200 nm (**c**), and 300 nm (**d**) of the AHM precursor, respectively.

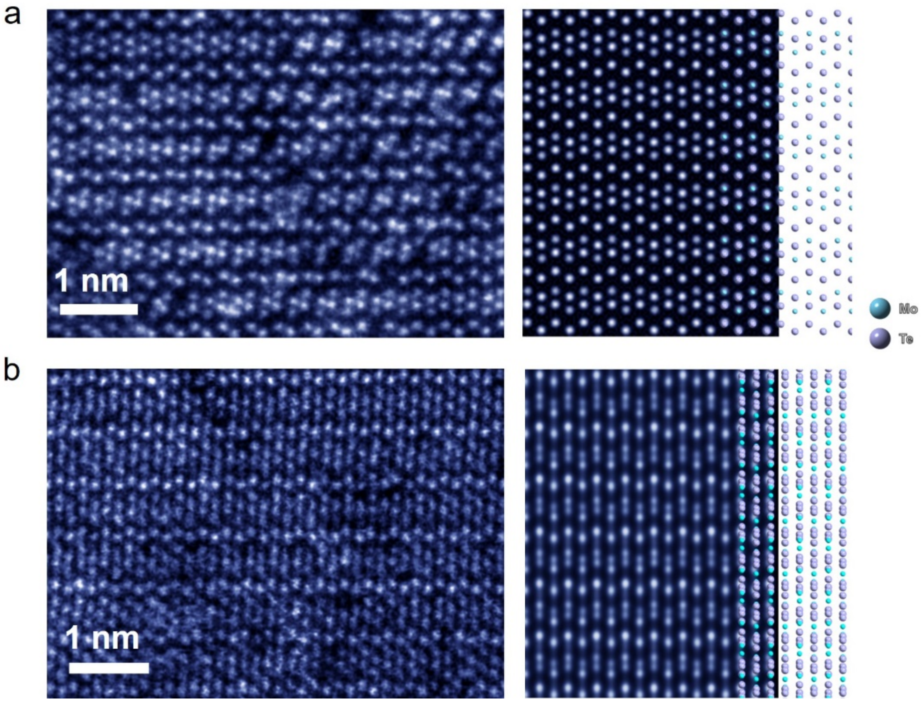

Supplementary Figure 4. ACSTEM images of monolayer and trilayer 1T′ MoTe₂. a,b, High-resolution atomic-scale HAADF image for monolayer and bilayer $MoTe_2$. The right panels indicate the corresponding simulated structures. The schematic monoclinic lattice and simulated image of monolayer and trilayer agree well with the HAADF image.

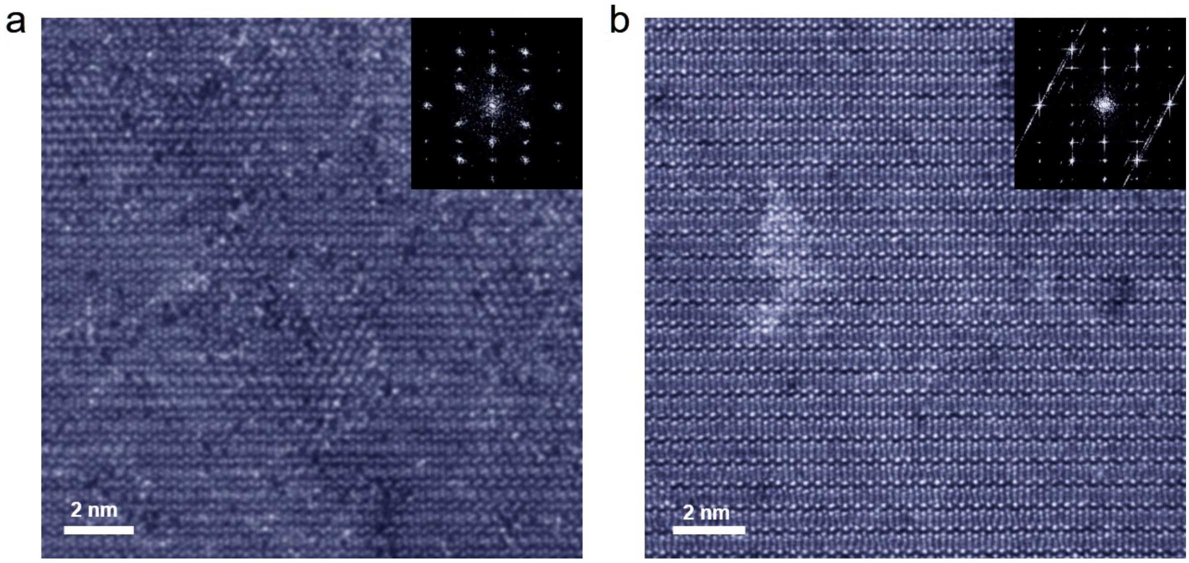

Supplementary Figure 5. Overview ACSTEM images of large-area 1T′ $MoTe_2$ layers. a,b, Large-scale atomic HAADF image of monolayer (**a**) and bilayer (**b**) $MoTe_2$ single crystals. Insets, the corresponding FFT patterns. The FFT patterns shown in the insets confirms the rectangular shape of the 1T′ $MoTe_2$ unit cell.

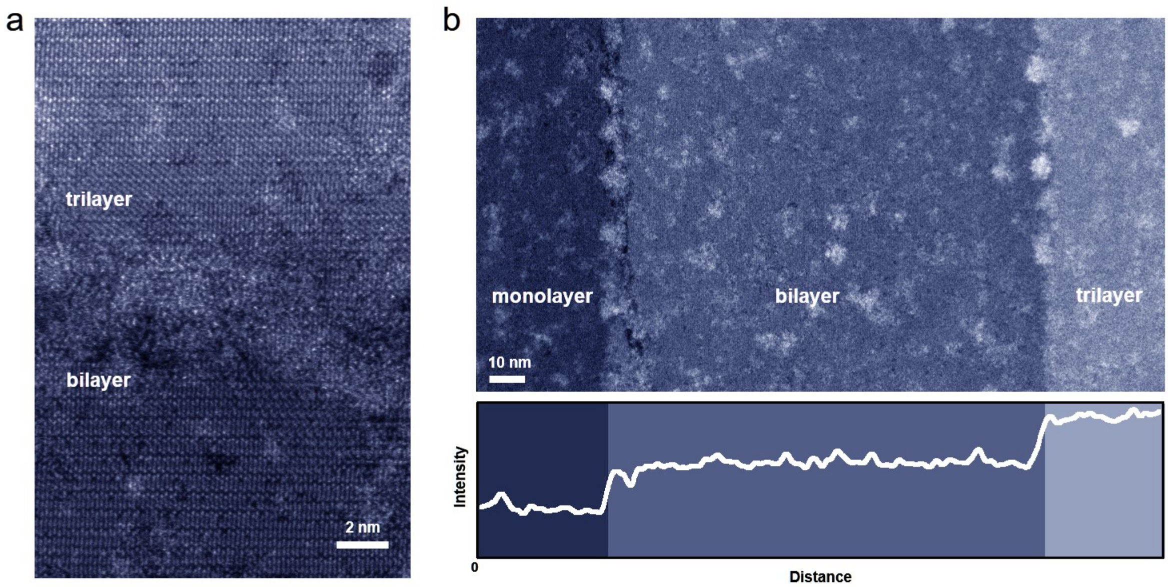

Supplementary Figure 6. ACSTEM images of 1T′ $MoTe_2$ with different layers. a, The interface of the bilayer and trilayer $MoTe_2$ single crystals. **b,** Low-magnification STEM images

of the $MoTe_2$ with monolayer, bilayer, and trilayer, showing that the brightness of trilayer and bilayer regions is roughly triple and double than that in the monolayer region, respectively.

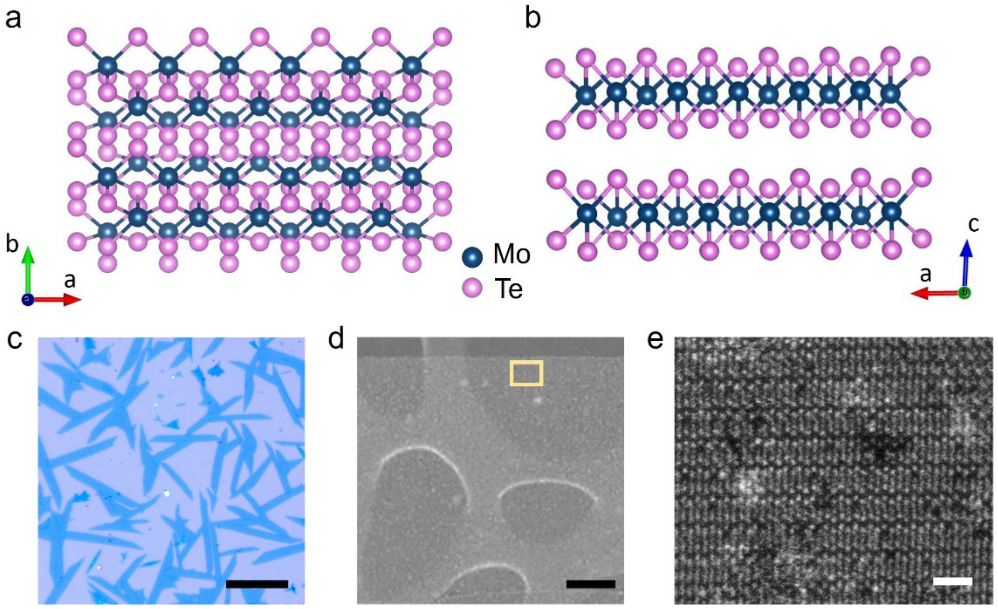

Supplementary Figure 7. Structural characterization of bilayer 1T′ $MoTe_2$ single crystals. **a**,**b**, Top (**a**) and side (**b**) views of the crystal structure of 1T′ $MoTe_2$. **c**, Optical image of bilayer 1T′ $MoTe_2$ single crystals. The scale bar is 50 μm. **d**, Low-magnification TEM image of a bilayer 1T′ $MoTe_2$ single crystal covering the TEM grids. The scale bar is 200 nm. **e**, High resolution STEM image showing the atomic structure with being 1T′ phase. The scale bar is 1 nm. As the CVD growth process is near equilibrium state, the zigzag chain direction is the most energy favourable edge[1]. The monoclinic crystal structure causes the morphology of the 1T′ $MoTe_2$ to be rectangular, and this has also been commonly observed[2-4].

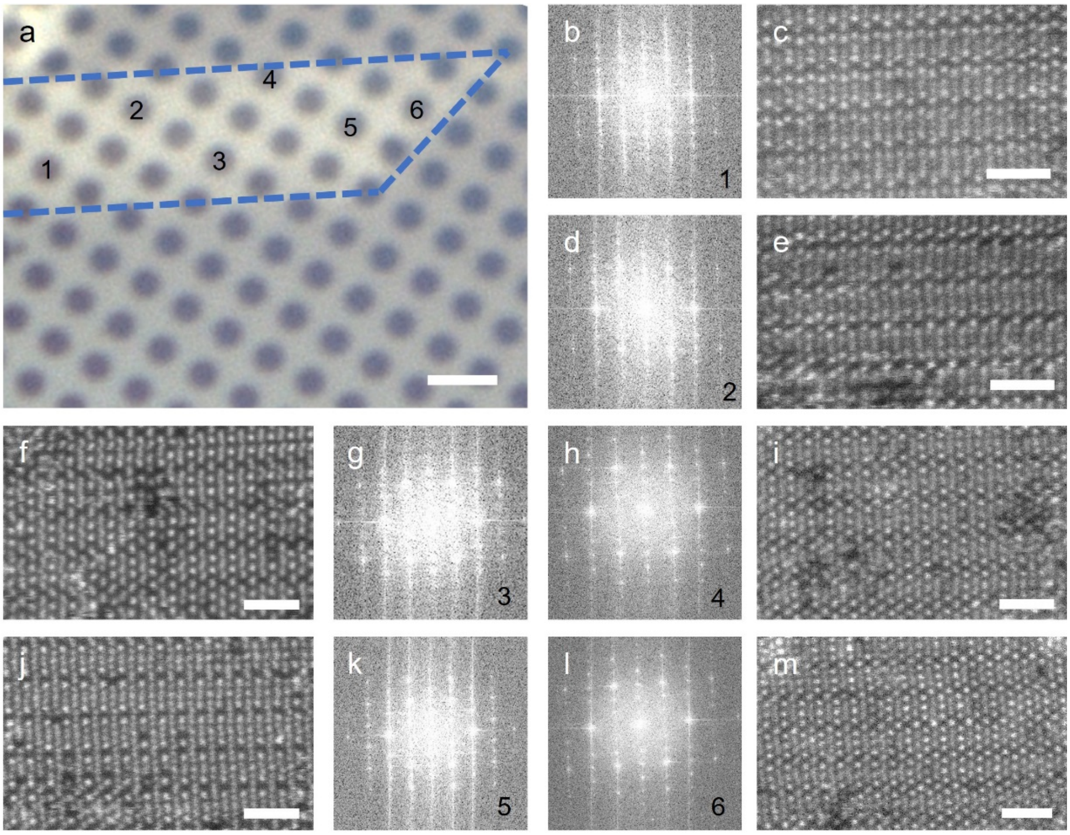

Supplementary Figure 8. STEM characterization of bilayer 1T′ $MoTe_2$ flake with different locations. a, Optical image of a rectangular bilayer $MoTe_2$ flake. The scale bar is 5 μm. **b-m**, Atomic STEM images (**c**,**e**,**f**,**i**,**j**,**m**) and corresponding FFT patterns (**b**,**d**,**g**,**h**,**k**,**l**) at different locations (1-6), respectively. All the scale bars are 1 nm. All the atomic arrangements show the same orientation, and the corresponding FFT show the same pattern, both of which confirm the single-crystal nature of the rectangular $MoTe_2$ flake showing the same orientation across the whole flake.

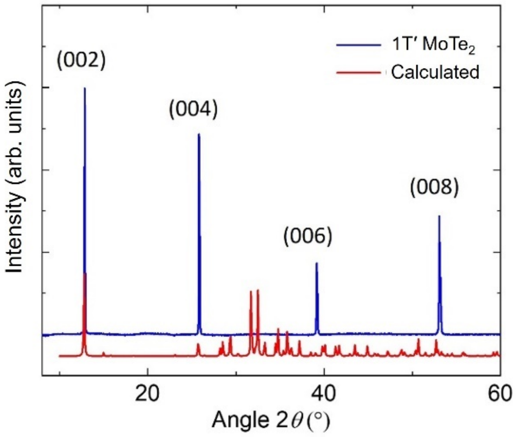

Supplementary Figure 9. XRD patterns of the large-scale 1T′ $MoTe_2$ films. As shown in the blue curve, there are only four main diffraction peaks corresponding to the (002), (004), (006), (008) planes of the $MoTe_2$ crystals, which is in good agreement with the reference pattern for monoclinic 1T′ phase. Obviously, all prominent diffraction peaks are indexed to the {001} family planes, suggesting that the *c*-axis of the as-grown film is perpendicular to the growth substrate and that the growth is highly textured.

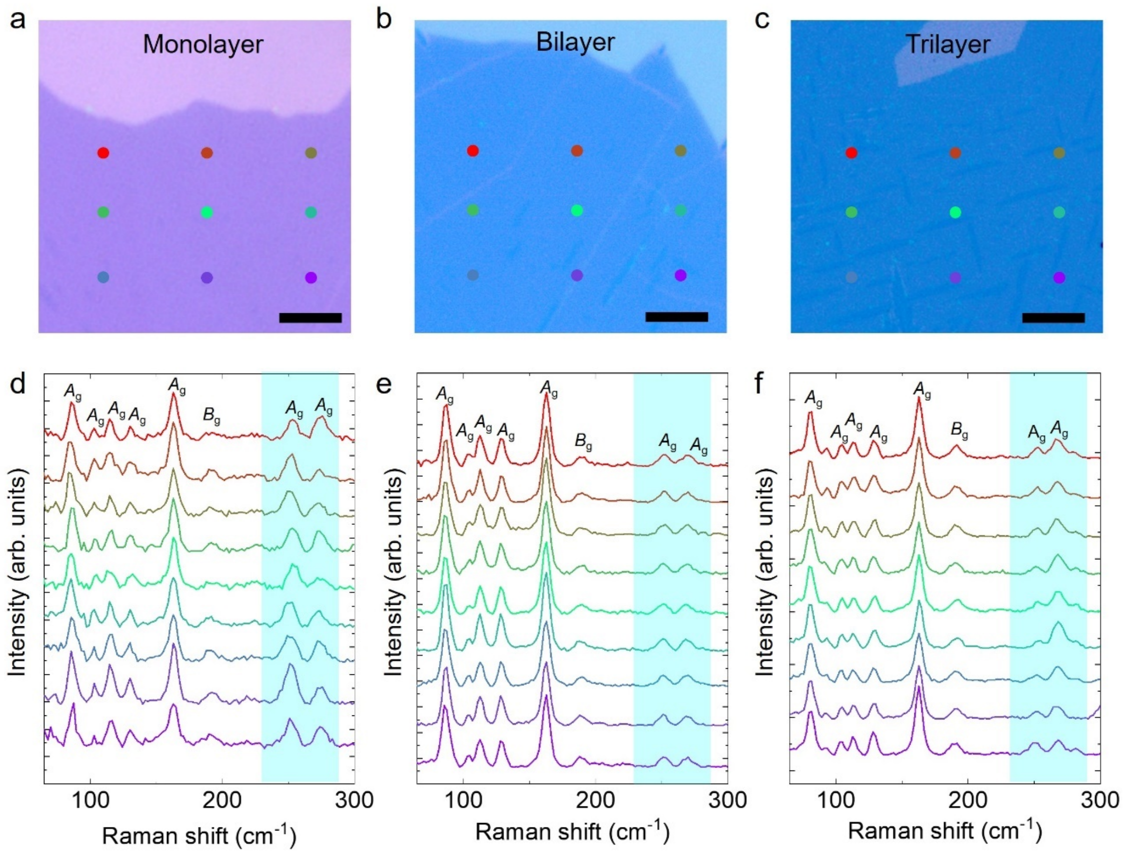

Supplementary Figure 10. Optical images and Raman spectra of large-scale $MoTe_2$ films with different layers. a-c, Optical image of large-scale monolayer (**a**), bilayer (**b**), and trilayer (**c**) $MoTe_2$ films. The scale bars in **a**, **b**, and **c** are 50 μm, 200 μm, and 200 μm, respectively. **d-f**, Raman spectra of $MoTe_2$ films, showing the monolayer (**d**), bilayer (**e**), and trilayer (**f**) feature in **a**, **b**, and **c**, respectively. The different coloured dots in **a**, **b**, and **c** indicate the positions acquired for Raman measurements. The excessively Raman measurements on monolayer, bilayer, and trilayer films show very similar peak characteristics, indicating the uniform feature.

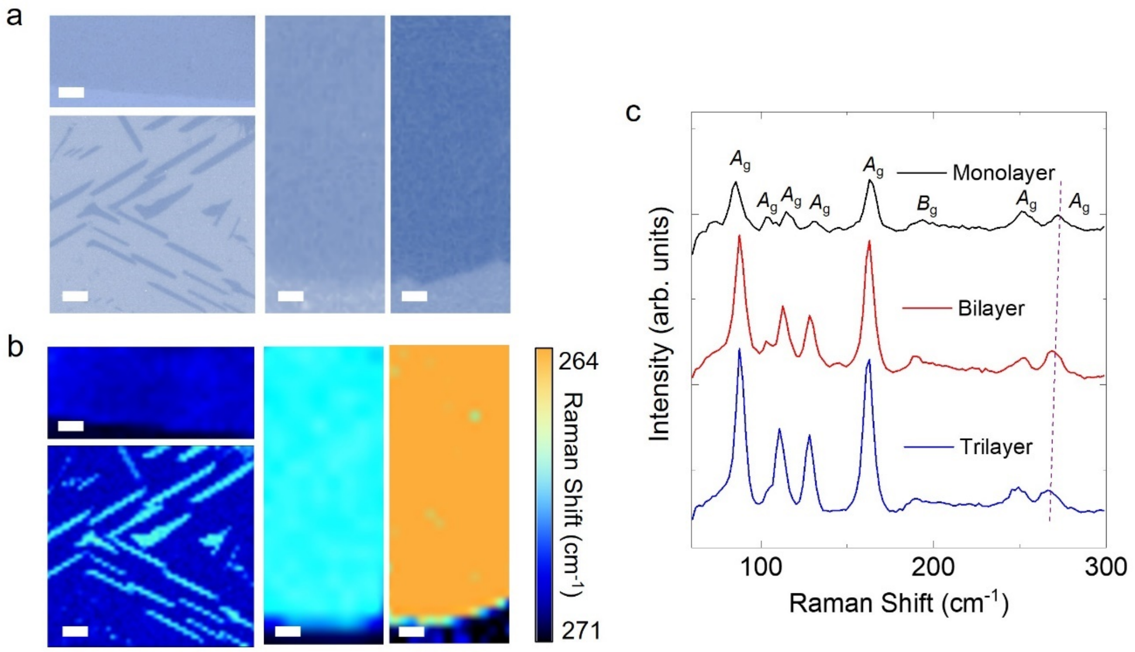

Supplementary Figure 11. Optical images and Raman characterization of $MoTe_2$ films with different layers. a,**b**, Optical image (**a**) and Raman mappings (**b**) of $MoTe_2$ monolayer (top left), second layer with different orientation on top of the monolayer (bottom left), bilayer film (middle), and trilayer films (right). All the scale bars are 2 μm. **c**, Typical Raman spectra of $MoTe_2$ with different layers, showing the 1T′ phase characteristics[5,6].

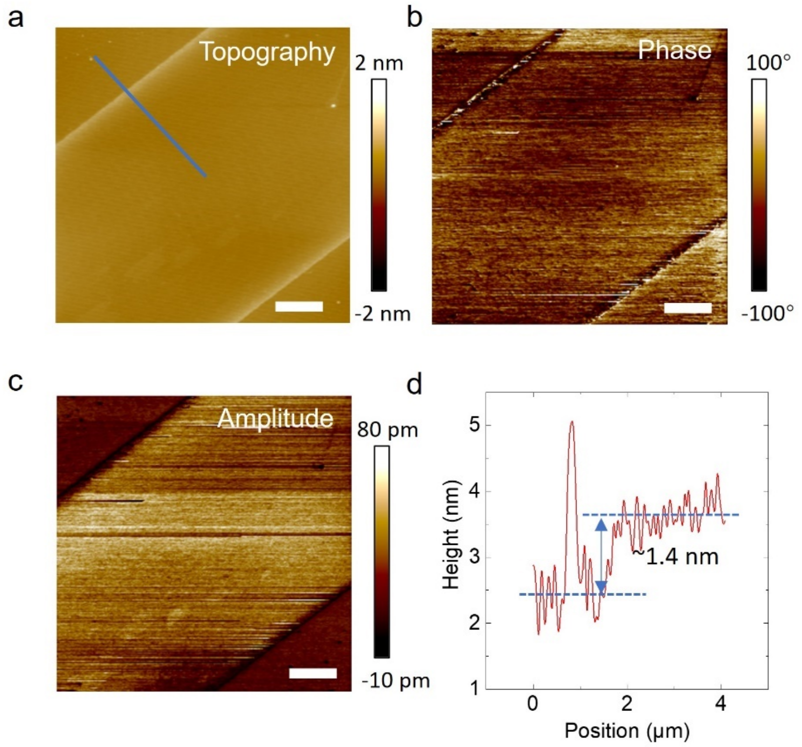

Supplementary Figure 12. PFM measurements on a bilayer 1T′ $MoTe_2$ single crystal. a-c, Topography (**a**), phase (**b**) and amplitude (**c**) of the bilayer 1T′ $MoTe_2$ single crystal before poling. All the scale bars are 2 μm. **d**, Height profile of the bilayer $MoTe_2$ single crystal, showing the height of ~1.4 nm.

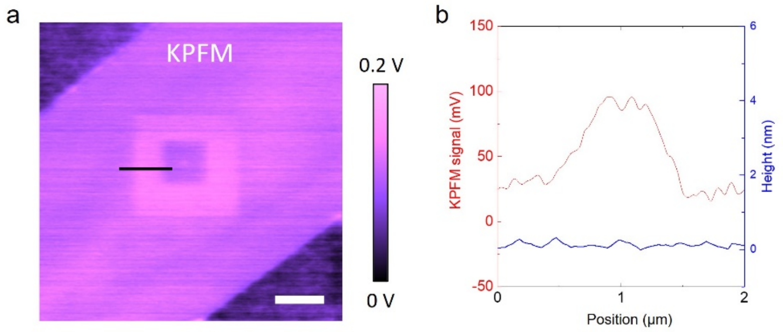

Supplementary Figure 13. Kelvin probe force microscope (KPFM) measurements on a polarized bilayer 1T′ $MoTe_2$ single crystal. a, KPFM images of the polarized area of the bilayer $MoTe_2$ single crystal, showing a box-in-box image. The scale bar is 2 μm. **b**, KPFM profile of the polarized area, showing a potential drop of 70 meV between the upward and downward polarization, indicating the real ferroelectric polarization.

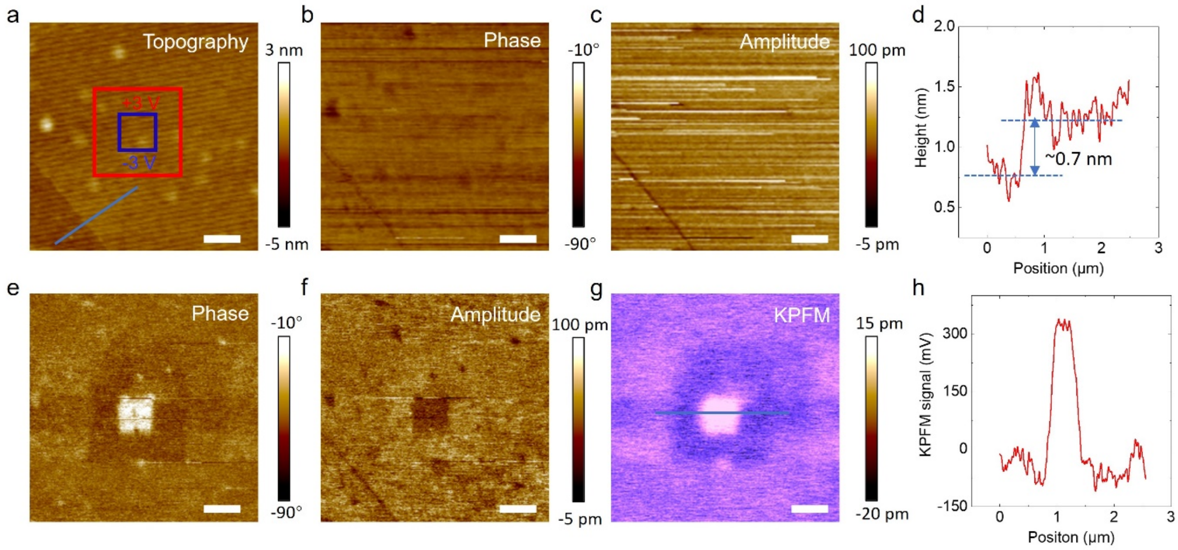

Supplementary Figure 14. PFM measurements on a monolayer 1T′ $MoTe_2$ single crystal. **a**-**c**, Topography (**a**), phase (**b**), and amplitude (**c**) of the initial monolayer 1T′ $MoTe_2$ single crystal. **d**, Height profile of the monolayer $MoTe_2$ showing the thickness of ~0.7 nm. **e**-**g**, Phase (**e**), amplitude (**f**), and KPFM image (**g**) of the monolayer $MoTe_2$ single crystal after applying DC bias +3 V in the central region of 5 μm followed by -3 V in the central region of 2 μm. **h**, Line profile in the KPFM signal (**g**) showing the potential drop for 500 mV after applying the voltage, indicating the phase change origins from the charge effect due to the highly nonconductive monolayer $MoTe_2$. All the scale bars are 2 μm.

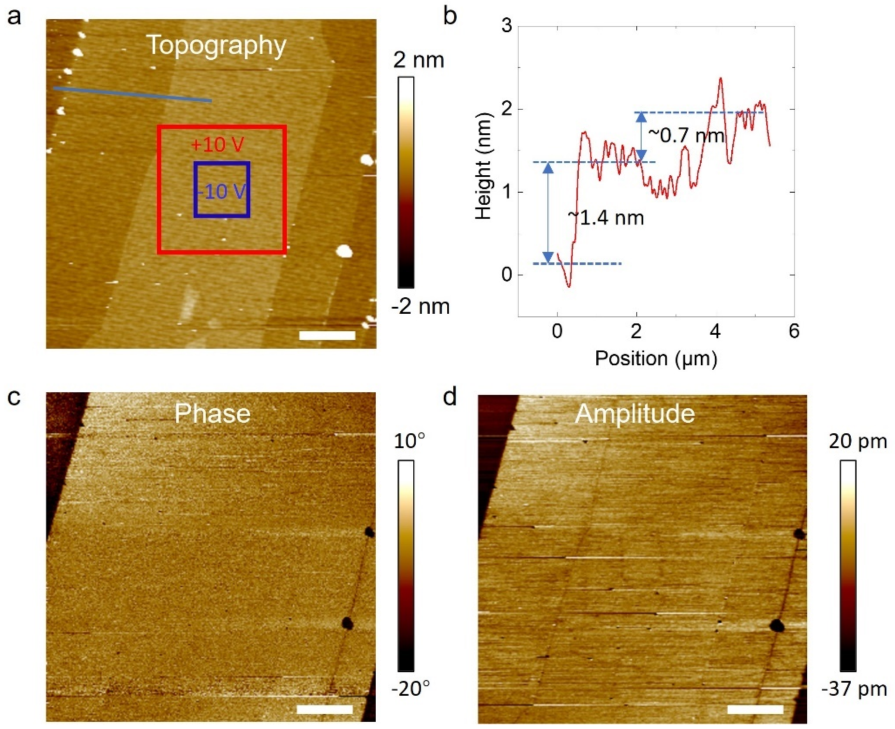

Supplementary Figure 15. PFM measurements on a trilayer 1T′ $MoTe_2$ single crystal. a,**b**, Topography (**a**) and line profile (**b**) show the thickness of the trilayer $MoTe_2$ is ~2.1 nm. **c**,**d**, Phase (**c**) and amplitude (**d**) of the trilayer $MoTe_2$ single crystal after applying DC bias +10 V in the central region of 5 μm followed by -10 V in the central region of 2 μm. All the scale bars are 2 μm. The neglectable phase change and slightly amplitude change indicates the trilayer $MoTe_2$ could not be switched.

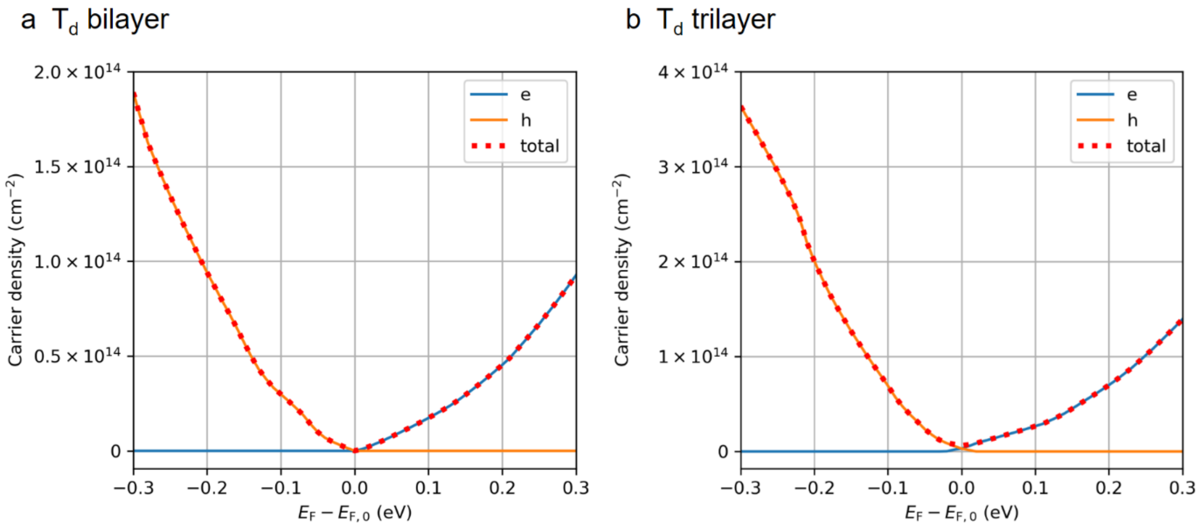

Supplementary Figure 16. Calculated carrier density on Fermi level in T_d bilayer (a) and trilayer $MoTe_2$ (b), respectively.

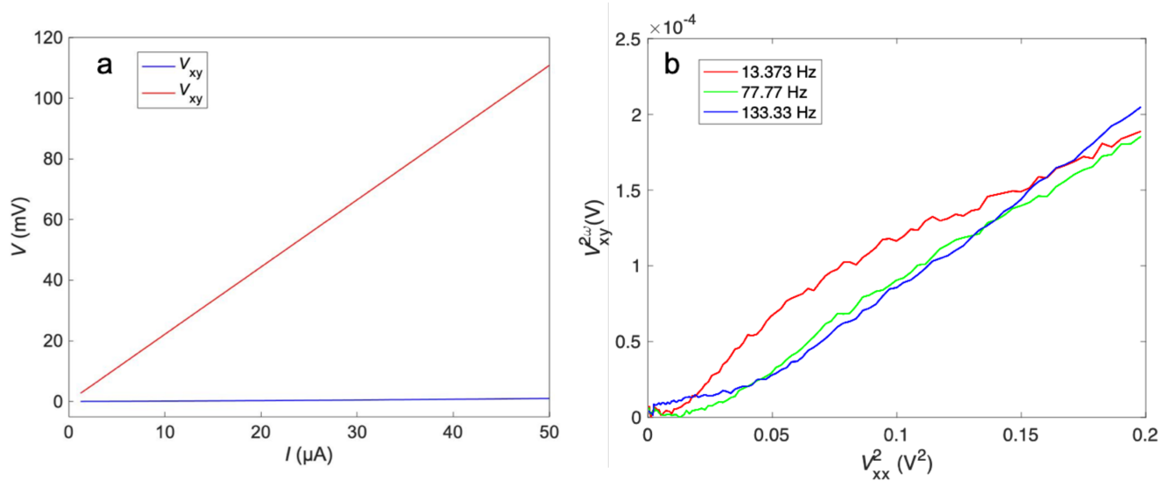

Supplementary Figure 17. Hall signals of bilayer $MoTe_2$ at 1.6 K. a, Hall voltage V_{xy} and longitudinal voltage V_{xx} are plotted vs current with blue and red curves, respectively. **b,** 2nd

harmonic signal measured at different frequencies. Frequency dependence of observed 2nd harmonic Hall signal is plotted in **b**. Second order signal at lock-in frequencies of 13.373 Hz, 77.77 Hz and 133.33 Hz are shown. The signals at different frequencies don't change significantly and remain in the same range. Thus, it excludes potential measurement artifacts such as spurious capacitive coupling.

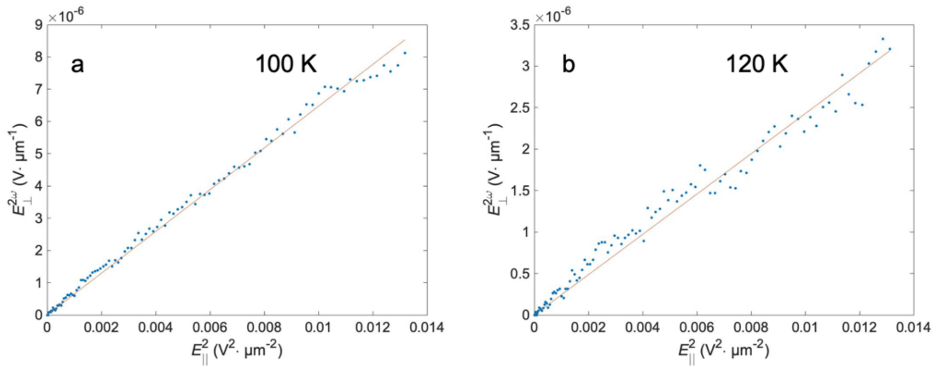

Supplementary Figure 18. Second order Hall signal as function of parallel electric field at 100 K (a) and 120 K (b) respectively. The 2nd order signal observed at temperatures higher than 100 K is substantially noisier and hence not used in analysis.

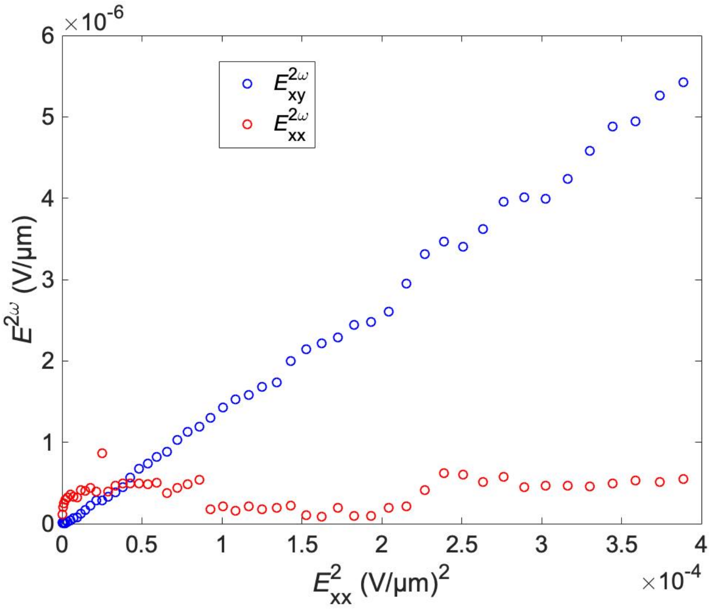

Supplementary Figure 19. Second order Hall (blue) and longitudinal (red) electric field as function of square of longitudinal electric field in a bilayer device.

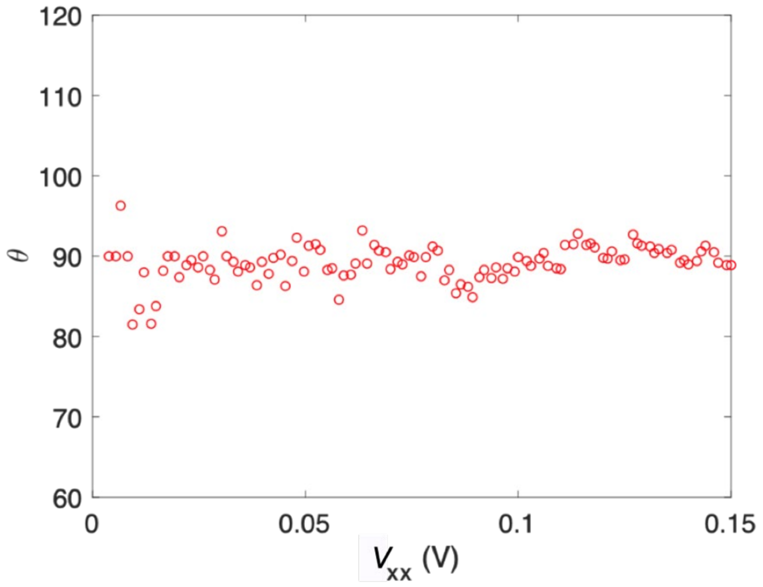

Supplementary Figure 20. Phase of 2nd order Hall signal as function of longitudinal voltage.

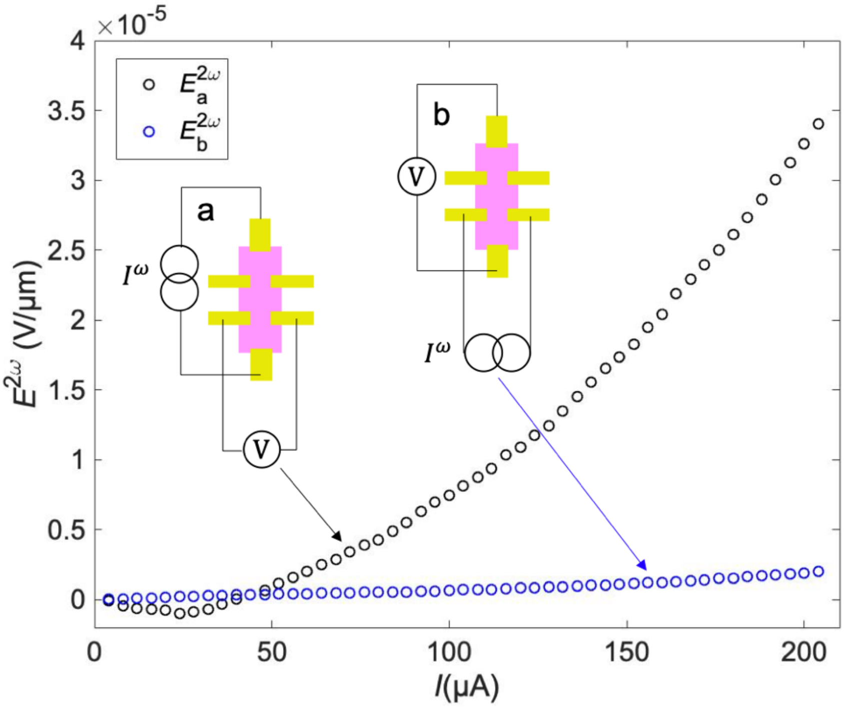

Supplementary Figure 21. Second order Hall electric field as function of applied current for normal geometry (black) and opposite geometry (blue), respectively. In the main text, we focus on the 2nd harmonic Hall signal where the current is applied along a-axis and we detected the voltage difference along b-axis (as denoted in the inset a). This measurement setup

is inspired by the theory that the Berry curvature dipole lies along a-axis due to mirror symmetry in $MoTe_2$. Here, we also measured the 2nd harmonic Hall signal in the opposite geometry. In this opposite geometry, the current passes along b-axis and we detected the voltage along *a*-axis (as denoted in the inset b). When plotted together, we can find the 2nd harmonic response acquired in opposite geometry is much weaker. This further confirms the symmetry selection rule.

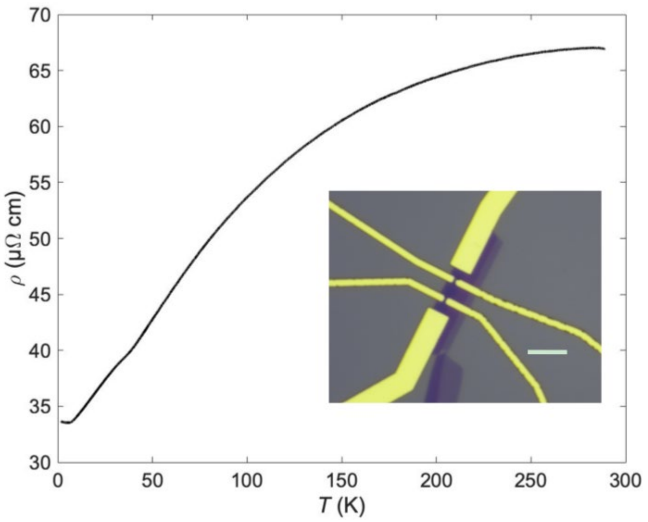

Supplementary Figure 22. Trilayer $MoTe_2$ resistivity as function of temperature. Inset: Hall bar contacts on trilayer $MoTe_2$. The scale bar is 5 μm.

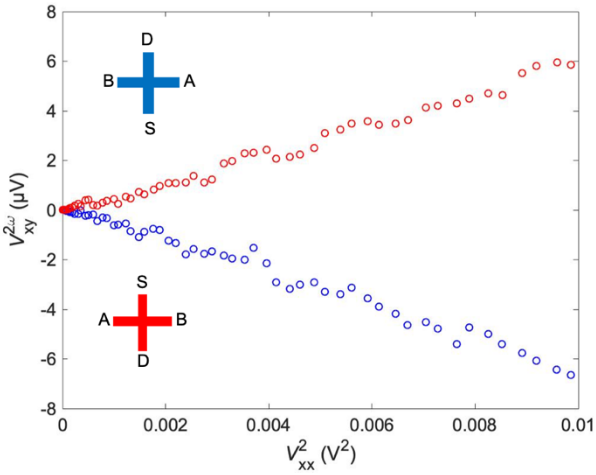

Supplementary Figure 23. Trilayer $MoTe_2$ 2nd harmonic Hall signal vs square of longitudinal voltage. Inset shows two opposite measurement setups. The linear dependence and sign reversal under two opposite measurement setups also consolidate the existence of 2nd harmonic response.

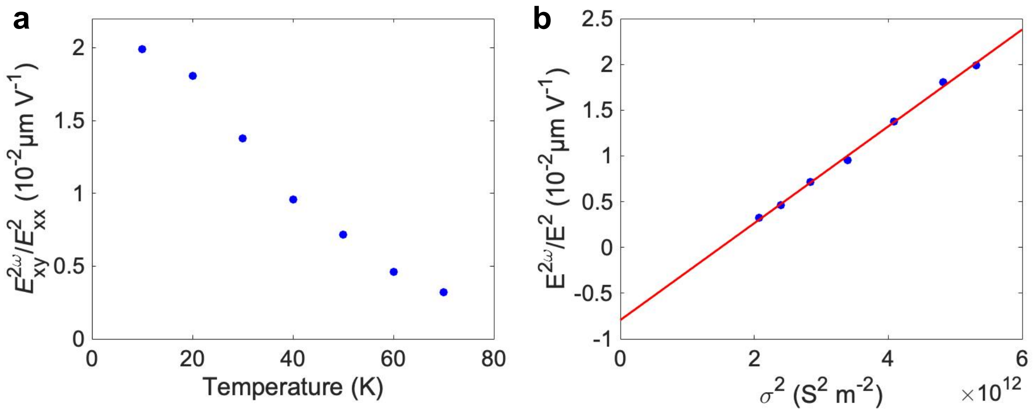

Supplementary Figure 24. Second harmonic Hall effect in 8-layer-thick $MoTe_2$. Second harmonic magnitude as function of temperature (**a**) and square of conductivity (**b**). The red line in **b** is linear fitting to the points. The smaller intercept $\sim 0.79 * 10^{-2}\mu\text{m V}^{-1}$ at Fermi energy of around 0.05 eV corresponds to a Berry curvature dipole of 0.047 nm. This value is one order of magnitude smaller than that of our CVD trilayer sample.

Supplementary Tables

Supplementary Table 1. Fitting parameters for bilayer sample. Parabolic curve fitting parameters of $\frac{E_{\perp}^{2\omega}}{E_{||}^{2}}$ dependence on conductivity σ_{xx} in Fig. 5b in the main text is shown in chart below.

Coefficient	2nd order	1st order	0th order (constant)
Value	-2.962e-13	2.465e-07	-0.044470

Supplementary Table 2: Nonlinear Hall electrical characteristics of different materials.

**Material**	**No. of dimensions**	**Conductivity range**	**Input current maximum(μA)**	**Output voltage maximum (μV)**	**NLH magnitude** $\frac{E_{xy}^{2\omega}}{E_{xx}^{2}}$ maximum ($\mu\text{m} \cdot \text{V}^{-1}$)	**Reference**
CVD $MoTe_2$ bilayer	2	$4 \sim 6 * 10^5\ \text{Sm}^{-1}$	97	125	7×10^{-3}	This work

CVD $MoTe_2$ trilayer	2	$1.8\sim2.5 * 10^6\ \mathrm{Sm^{-1}}$	93	6.5	1.2×10^{-2}	This work
8-layer exfoliated $MoTe_2$	2	$1.4\sim2.3 * 10^6\ \mathrm{Sm^{-1}}$	810	5.6	2×10^{-2}	This work
Bilayer WTe_2	2	$\sim6 * 10^5\ \mathrm{Sm^{-1}}$	1	170	1.4×10^{-1}	Ref[7]
Few-layer WTe_2	2	$3\sim6 * 10^5\ \mathrm{Sm^{-1}}$	600	30	1×10^{-3}	Ref[8]
Strained monolayer WSe_2	2	$2 * 10^6\ \mathrm{Sm^{-1}}$	4.5	20	6.2×10^{-1}	Ref[9]
Corrugated bilayer graphene	2	$\sim5 * 10^5\ \mathrm{Sm^{-1}}$	0.1	1	NA	Ref[10]
Bi_2Se_3 surface	2	$\sim1.1 * 10^5\ \mathrm{Sm^{-1}}$	1500	20	0.8×10^{-3}	Ref[11]
Bulk WTe_2	3	$10^7\ \mathrm{Sm^{-1}}$	4000	2	NA	Ref[12]
Cd_3As_2	3	$4 * 10^7\ \mathrm{Sm^{-1}}$	4000	1	NA	Ref[12]
$Ce_3Bi_4Pd_3$	3	$10^7\ \mathrm{Sm^{-1}}$	6300	40	NA	Ref[13]
$TaIrTe_4$	3	$10^6\ \mathrm{Sm^{-1}}$	600	120	2×10^{-2}	Ref[14]
T_d $MoTe_2$ (c-axis)	3	$\sim10^7\ \mathrm{Sm^{-1}}$	5000	1	10^5	Ref[15]
α-(BEDT-TTF)$_2I_3$	3	$10^3\ \mathrm{Sm^{-1}}$	1000	10	NA	Ref[16]

Supplementary Notes

Supplementary Note 1. Discussion on Layer-dependent Raman spectroscopy.

Raman spectroscopy is a powerful tool to investigate the crystal symmetry, interlayer coupling and layer stacking in 2D materials. For 1T′ $MoTe_2$, it has several characteristic Raman peaks: a prominent peak of A_g mode at ≈ 85 cm^{-1}, two A_g modes at ≈ 104 cm^{-1} and ≈ 116 cm^{-1}, another prominent A_g mode at ≈ 162 cm^{-1}, a B_g mode at ≈ 190 cm^{-1}, and two A_g modes at ≈ 258 cm^{-1} and ≈ 269 cm^{-1}. These Raman features are consistent with theoretical predictions and Raman spectra of exfoliated 1T′ $MoTe_2$[2]. For the Raman mode at low frequency, the A_g peak at ≈ 13 cm^{-1} would appear for bilayer and trilayer samples[5]. The monolayer $MoTe_2$ could be stacked in two forms: one is 1T′ stacking and the other is T_d stacking. The 1T′ and T_d $MoTe_2$ can be differentiated in interlayer vibration modes, in which the A_g mode at ≈ 116 $cm^{-1}$and the B_g mode at ≈ 190 cm^{-1} in 1T′ $MoTe_2$ are split into two peaks in T_d phase. Based on this, our CVD-grown $MoTe_2$ shows clearly the characteristics of 1T′ phase, which is also in agreement with the STEM observations. For the layer numbers, the higher energy mode at ≈ 271 cm^{-1} red shifts when the thickness of 1T′ $MoTe_2$ increase from monolayer to bilayer (268 cm^{-1}), and trilayer (265 cm^{-1}). This is because the out-of-plane A_g mode at this energy mode is strongly affected by the interlayer interactions. It is considered that such a frequency drop with increased thickness is possibly due to the enhancement of dielectric screening of the long-range Coulomb interaction in thicker $MoTe_2$[6]. Therefore, a fingerprint Raman peak at 269 cm^{-1} distinguishes monolayer $MoTe_2$ and the peak position redshifts to 267, and 265 cm^{-1} for bilayer and trilayer, respectively. As shown in Supplementary Figure 11, the Raman mapping of this peak shows a very uniform signal at 269 cm^{-1} for monolayer $MoTe_2$ film (top left), 267 cm^{-1} for bilayer crystals with different orientations (bottom left) and film (middle panel), and 265 cm^{-1} for trilayer films (right panel), suggesting the uniform feature for the $MoTe_2$ with different layers.

Supplementary Note 2. Density functional theory calculations.

From the calculation of band structures, carrier density depending on the Fermi level change can be obtained as shown in Supplementary Figure 16 by integrating their occupation numbers. Carrier numbers per unit cell are $N_e = \sum_{n\in con}[ab/(2\pi)^2]\int d^2\mathbf{k}\, f_0(E_{n\mathbf{k}}, E_F, T)$ for electrons and $N_h = \sum_{n\in val}[ab/(2\pi)^2]\int d^2\mathbf{k}[1 - f_0(E_{n\mathbf{k}}, E_F, T)]$ for holes. n ∈ con (val) means the band index belonging to the conduction (valence) band. f_0 is Fermi-Dirac distribution. T= 0 K is assumed in this calculation.

The band structures can explain the existence of the peaks in the Berry curvature dipole in terms of the crossing and anti-crossing points among different bands. They tend to be a source of the Berry curvature so that the corresponding peaks appear in the Berry curvature dipole for

the crossing point[17] and anti-crossing point[7]. Since the experiment identified heavy electron doping, we mainly focused on the crossing/anti-crossing points in the conduction bands.

The Berry curvature distributions were calculated in two different schemes, fixed-Fermi-level scheme (Fig. 3c and e) and fixed-number-of-occupied-bands scheme (Fig. 3d and f). In the fixed-Fermi-level scheme, the number of occupied bands at each **k**-point is determined by the fixed Fermi level, as it is usually done, and the Berry curvature is summed over the corresponding occupied bands. Therefore, the Berry curvature tends to change discontinuously at the Fermi line at which the number of occupied bands changes. This scheme is a proper definition which gives the proper distribution for the physical quantities such as the Berry curvature dipole at the corresponding Fermi level. In the fixed-number-of-occupied-bands scheme, the number of occupied bands is fixed regardless of the band energy levels and the Fermi level. This scheme is useful when we need to highlight the Berry curvature from a specific band structure such as crossing/anti-crossing point. WANNIERTOOLS code adopts this scheme[18]. In our case, the lowest and the second lowest conduction bands were considered as the highest occupied band for the bilayer and trilayer, respectively.

It is noteworthy that the crossing points of the T_d-bilayer are not the Weyl points, which is reported in the bulk $MoTe_2$[19]. Weyl points always exist as a pair of a source and a sink of the Berry curvature. However, in the bilayer, the two crossing points represent a pair of two Berry curvature sources.

In our case, peaks in the Berry curvature dipole are far below the Fermi level due to doping. Therefore, the corresponding Berry curvature dipole is much smaller than the peak values. However, one should note that the peak level may depend also on the details of the calculation, e.g., the HSE parameter.

Supplementary Note 3. Device fabrication and encapsulation

The as-grown $MoTe_2$ on SiO_2/Si wafers are instantly transferred into argon-filled glovebox after CVD growth. Large rectangular bilayer (trilayer) $MoTe_2$ flakes are examined under optical microscope. E-beam resist is subsequently spin-coated and sample is pre-exposure baked. Standard e-beam lithography process and e-beam evaporation process is done. After that sample is transferred into the glovebox for liftoff, a thin (20-40 nm) hexagonal boron nitride flake is transferred on top to encapsulate sample and prevent degradation.

Supplementary Note 4: Electrical characteristics of bilayer $MoTe_2$

A rectangular bilayer $MoTe_2$ Hall bar sample has only crystalline symmetry which is the mirror plane M_a. Thus an AC current along a-axis would cause a 2nd harmonic Hall response along b-axis. Immediately after fabrication, the device was loaded in the Oxford Teslatron system. AC measurements were conducted with lock-in instruments (SR830). All measurements were conducted at base temperature 1.6 K unless otherwise stated.

First order Hall measurement without magnetic field is shown in the left panel of Supplementary Figure 17. The V_{xx}'s linear longitudinal dependence on current demonstrates the Ohmic behavior. The first order Hall signal V_{xy} is less than 1% of measured longitudinal signal. The observed Hall signal could originate from tiny misalignment of electrodes or intrinsic resistance anisotropy of material.

During cooling down, the resistance of bilayer $MoTe_2$ shows an upswing at low temperatures. The upswing in resistance at low temperature for few-layer samples has been reported previously[20-22]. The transport behavior of WTe_2 goes through metal-insulator transition when the vertical thickness of the sample is reduced[20]. The authors found that the mobility of the charge carriers are 2-3 orders of magnitude smaller than that of thicker layers and thus disorder should be the cause. They argue that the carriers are Anderson localized. The metal-insulator transition in few-layer $MoTe_2$ is also explained as enhanced charge carrier localization[21].

We have also noticed a report that even monolayer $MoTe_2$ shows metallic behavior down to low temperature[23]. The main difference is that the authors used hBN to fully encapsulate the $MoTe_2$ flake. In our case, our sample was only covered by hBN on top to provide protection from degradation and the SiO_2/Si substrate has an influence on $MoTe_2$. In surveying literature, we found that the substrate influences the device behavior of the $MoTe_2$ sample significantly, in which few-layer $MoTe_2$ has been reported to exhibit insulating or 'gapped/band-splitting' behaviors depending on the substrate. For instance, the R-T relation of the 2 nm-thick $MoTe_2$ (can be considered as bilayer) also shows an upswing below 50 K before transition into superconducting state[22]. We observed that the authors used SiO_2/Si substrate similar to ours. Additionally, monolayer $MoTe_2$ exhibit semi-metallic behavior with large band overlap when grown on bilayer graphene[24] but a weak overlap with a potential gap-opening when exfoliated on gold substrates[25]. Therefore, we can infer that the electronic band structure of few-layer $MoTe_2$ is very sensitive to the substrate. Based on the above analysis, our bilayer sample may have more skew scattering from SiO_2/Si substrate compared to hBN. This point is also reflected in our discussion of the NLH magnitude in the paper in which we stated that the NLE has multiple contributions (i.e., skew scattering etc), and not limited to intrinsic Berry curvature. Due to low conductivity of the bilayer, the relation of NLH magnitude with conductivity is

complex and cannot be quantified analytically at the present stage. In contrast, for metallic trilayer $MoTe_2$, the linear relation in Fig. 5d clearly agrees well with both theory and published literatures.

Apart from the 2nd order Hall response, we have also measured the 2nd order longitudinal response. A comparison of these two responses plotted against longitudinal electric field is shown in Supplementary Figure 19. It is clearly shown that the 2nd order Hall signal dominates over the longitudinal counterpart, which also rules out the possibility of thermal-induced 2nd order effect which is isotropic. Also, we observed that the phase of 2nd Hall voltage with respect to longitudinal voltage has a 90-degree phase shift, as shown in Supplementary Figure 20. The consistent phase further supports the 2nd order origin since a straightforward relation of 2nd harmonic signal should follow as:

$$V^{\text{second-order}} \propto [I_0 \sin(\omega t)]^2 = I_0^2[1 + \sin(2\omega t - \pi/2)]/2 \qquad \text{Eq. (1)}$$

Where the 2nd order part should have a 90-degree phase shift with respect to applied voltage.

Supplementary Note 5. Electrical characteristics of trilayer $MoTe_2$

Trilayer CVD-grown $MoTe_2$ devices are also fabricated and measured in much similar approach to provide a contrastive counterpart. In general, trilayer samples show metallic behavior in resistivity-T measurement (Supplementary Figure 22). This metallic behavior is consistent with previous reports[26,27], which consolidates the quality of our grown samples.

For Fig. 5d in the main text, we can observe linear dependence relation in thicker samples[8,15].

$$\frac{E_{\perp}^{2\omega}}{E_{\|}^{2}} = \alpha \cdot \sigma^2 + \beta \qquad \text{Eq. (2)}$$

We can also rewrite the NLH magnitude in terms of anomalous/longitudinal conductivity ratio

$$\frac{E_{\perp}^{2\omega}}{E_{\|}^{2}} = \frac{\sigma_{\text{AH}}}{r\sigma}\frac{1}{E_{\|}} \qquad \text{Eq. (3)}$$

here $r \equiv \frac{\rho_{\text{a}}}{\rho_{\text{b}}}$ is the anisotropic resistance ratio of around 0.37 from our experiment.

In low-frequency limit, as in our experiment, the intrinsic contribution to the nonlinear Hall conductivity[28] can be written as $\sigma = \frac{\pi}{2} k \frac{G_0}{d} D$, where D is Berry curvature dipole, G_0 is the conductance quantum and $\hbar k \equiv eE_{\|}\tau$ is the net electron momentum obtained under an in-plane bias $E_{\|}$ ($\hbar$ and e denote, Planck constant electron charge). The longitudinal conductivity

can be expressed as $\sigma = G_0 v_F k_F \tau / 2d$, where v_F and k_F are the Fermi velocity and Fermi vector, respectively, and are related to the Fermi energy as $\epsilon \sim \hbar v_F k_F$. We can thus obtain $\frac{\sigma_{AH}}{\sigma E_{||}} \sim \frac{\pi D e}{\hbar v_F k_F} \sim \frac{\pi D}{\epsilon_F / e}$.

In this relation, the dipole can be evaluated from the experimental value of $\frac{\sigma_{AH}}{\sigma E_{||}}$ in the limit of $\sigma \to 0$ where the only contribution comes from intrinsic effect Berry curvature dipole D (neglect side jump). Thus, a linear fit to curve in Fig. 5d can yield y-axis intercept $\beta \sim 1.34 * 10^{-2} \mu\mathrm{m\,V}^{-1}$, which scales directly with Berry curvature dipole D if we neglect the contribution from side jump.

$$D \sim \beta \cdot \frac{\epsilon_F}{e} \cdot \frac{r}{\pi} \qquad \text{Eq. (4)}$$

With carrier density determined from Hall measurement ($\sim 1 \times 10^{14}\,\mathrm{cm}^{-2}$), which corresponds to Fermi level at ~0.24 eV, then we can obtain Berry curvature dipole ~0.3 nm. This value is comparable to values reported for bilayer WTe_2 recently[7], but one order of magnitude larger than that of few-layer WTe_2[8].

Supplementary References

1 Zhu, D. *et al.* Capture the growth kinetics of CVD growth of two-dimensional MoS_2. *npj 2D Mater. Appl.* **1**, 8 (2017).

2 Naylor, C. H. *et al.* Monolayer single-crystal 1T'-$MoTe_2$ grown by chemical vapor deposition exhibits weak antilocalization effect. *Nano Lett.* **16**, 4297-4304 (2016).

3 Sung, J. H. *et al.* Coplanar semiconductor-metal circuitry defined on few-layer $MoTe_2$ via polymorphic heteroepitaxy. *Nat. Nanotech.* **12**, 1064-1070 (2017).

4 Pace, S. *et al.* Synthesis of large-scale monolayer 1T'-$MoTe_2$ and its stabilization via scalable hBN encapsulation. *ACS Nano* **15**, 4213-4225 (2021).

5 Cheon, Y., Lim, S. Y., Kim, K. & Cheong, H. Structural phase transition and interlayer coupling in few-layer 1T' and T_d $MoTe_2$. *ACS Nano* **15**, 2962-2970 (2021).

6 Zhou, L. *et al.* Sensitive phonon-based probe for structure identification of 1T' $MoTe_2$. *J. Am. Chem. Soc.* **139**, 8396-8399 (2017).

7 Ma, Q. *et al.* Observation of the nonlinear Hall effect under time-reversal-symmetric conditions. *Nature* **565**, 337-342 (2019).

8 Kang, K., Li, T., Sohn, E., Shan, J. & Mak, K. F. Nonlinear anomalous Hall effect in few-layer WTe_2. *Nat. Mater.* **18**, 324-328 (2019).

9 Qin, M. -S. *et al.* Strain tunable Berry curvature dipole, orbital magnetization and nonlinear Hall effect in WSe_2 monolayer. *Chin. Phys. Lett.* **38**, 017301 (2021).

10 Ho, S.-C. *et al.* Hall effects in artificially corrugated bilayer graphene without breaking time-reversal symmetry. *Nat. Electron.* **4**, 116-125 (2021).

11 He, P. *et al.* Quantum frequency doubling in the topological insulator Bi_2Se_3. *Nat. Commun.* **12**, 698 (2021).

12 Shvetsov, O. O., Esin, V. D., Timonina, A. V., Kolesnikov, N. N. & Deviatov, E. V. Nonlinear Hall effect in three-dimensional Weyl and dirac semimetals. *JETP Lett.* **109**, 715-721 (2019).

13 Dzsaber, S. *et al.* Giant spontaneous Hall effect in a nonmagnetic Weyl–Kondo semimetal. *Proc. Natl. Acad. Sci. U. S. A.* **118**, e2013386118 (2021).

14 Kumar, D. *et al.* Room-temperature nonlinear Hall effect and wireless radiofrequency rectification in Weyl semimetal $TaIrTe_4$. *Nat. Nanotech.* **16**, 421-425 (2021).

15 Tiwari, A. *et al.* Giant c-axis nonlinear anomalous Hall effect in T_d-$MoTe_2$ and WTe_2. *Nat. Commun.* **12**, 2049 (2021).

16 Kiswandhi, A. & Osada, T. Observation of possible nonlinear anomalous Hall effect in organic two-dimensional Dirac fermion system. *J. Condens. Matter Phys*. **34**, 105602 (2021).

17 Zhang, Y., Sun, Y. & Yan, B. Berry curvature dipole in Weyl semimetal materials: An ab initio study. *Phys. Rev. B* **97**, 041101 (2018).

18 Wu, Q., Zhang, S., Song, H.-F., Troyer, M. & Soluyanov, A. A. WannierTools: An open-source software package for novel topological materials. *Comput. Phys. Commun.* **224**, 405-416 (2018).

19 Sun, Y., Wu, S.-C., Ali, M. N., Felser, C. & Yan, B. Prediction of Weyl semimetal in orthorhombic $MoTe_2$. *Phys. Rev. B* **92**, 161107 (2015).

20 Wang, L. *et al.* Tuning magnetotransport in a compensated semimetal at the atomic scale. *Nat. Commun.* **6**, 8892 (2015).

21 Song, P. *et al.* Few-layer 1T′ $MoTe_2$ as gapless semimetal with thickness dependent carrier transport. *2D Mater.* **5**, 031010 (2018).

22 Cui, J. *et al.* Transport evidence of asymmetric spin-orbit coupling in few-layer superconducting $1T_d$-$MoTe_2$. *Nat. Comm.* **10**, 2044 (2019).

23 Rhodes, D. A. *et al.* Enhanced superconductivity in monolayer T_d-$MoTe_2$. *Nano Lett.* **21**, 2505-2511 (2021).

24 Tang, S. *et al.* Electronic structure of monolayer 1T′-$MoTe_2$ grown by molecular beam epitaxy. *APL Mater.* **6**, 026601 (2018).

25 Pawlik, A. S. *et al.* Thickness dependent electronic structure of exfoliated mono- and few-layer 1T′−$MoTe_2$. *Phys. Rev. Mater.* **2**, 104004 (2018).

26 Fatemi, V. *et al.* Magnetoresistance and quantum oscillations of an electrostatically tuned semimetal-to-metal transition in ultrathinWTe_2. *Phys. Rev. B* **95**, 041410(R) (2017).
27 Fei, Z. Y. *et al.* Edge conduction in monolayer WTe_2. *Nat. Phys.* **13**, 677-682 (2017).
28 Sodemann, I. & Fu, L. Quantum nonlinear Hall effect induced by Berry curvature dipole in time-reversal invariant materials. *Phys. Rev. Lett.* **115**, 216806 (2015).
